# The Development of a Digital Patient Navigation Tool to Increase Colorectal Cancer Screening Among Federally Qualified Health Center Patients: Acceptability and Usability Testing

**DOI:** 10.2196/53224

**Published:** 2024-09-25

**Authors:** Leah C Savage, Luz Estefhany Soto-Cossio, Francesca Minardi, Matthew Beyrouty, Julie Schoonover, Jay Musella, Michaela Frazier, Cristina N Villagra, Jamilia R Sly, Joel Erblich, Steven H Itzkowitz, Lina H Jandorf, Neil S Calman, Ashish Atreja, Sarah J Miller

**Affiliations:** 1 Department of Population Health Science and Policy Icahn School of Medicine at Mount Sinai New York, NY United States; 2 The Institute for Family Health New York, NY United States; 3 Department of Family Medicine and Community Health Icahn School of Medicine at Mount Sinai New York, NY United States; 4 Department of Psychology Hunter College City University of New York New York, NY United States; 5 Division of Gastroenterology Department of Medicine Icahn School of Medicine at Mount Sinai New York, NY United States; 6 Innovation Technology Division University of California Davis Sacramento, CA United States

**Keywords:** digital navigation, digital health, Federally Qualified Health Center, colorectal cancer, cancer screening, mobile phone

## Abstract

**Background:**

Federally Qualified Health Centers (FQHCs) are an essential place for historically underserved patients to access health care, including screening for colorectal cancer (CRC), one of the leading causes of cancer death in the United States. Novel interventions aimed at increasing CRC screening completion rates at FQHCs are crucial.

**Objective:**

This study conducts user testing of a digital patient navigation tool, called eNav, designed to support FQHC patients in preparing for, requesting, and completing CRC screening tests.

**Methods:**

We recruited English- and Spanish-speaking patients (N=20) at an FQHC in New York City to user-test the eNav website (2 user tests; n=10 participants per user test). In each user test, participants engaged in a “think aloud” exercise and a qualitative interview to summarize and review their feedback. They also completed a baseline questionnaire gathering data about demographics, technology and internet use, medical history, and health literacy, and completed surveys to assess the website’s acceptability and usability. Based on participant feedback from the first user test, we modified the eNav website for a second round of testing. Then, feedback from the second user test was used to modify and finalize the eNav website.

**Results:**

Survey results supported the overall usability and acceptability of the website. The average System Usability Scale score for our first user test was 75.25; for the second, it was 75.28. The average Acceptability E-scale score for our first user test was 28.3; for the second, it was 29.2. These scores meet suggested benchmarks for usability and acceptability. During qualitative think-aloud exercises, in both user tests, many participants favorably perceived the website as motivating, interesting, informative, and user-friendly. Respondents also gave suggestions on how to improve the website’s content, usability, accessibility, and appeal. We found that some participants did not have the digital devices or internet access needed to interact with the eNav website at home.

**Conclusions:**

Based on participant feedback on the eNav website and reported limitations to digital access across both user tests, we made modifications to the content and design of the website. We also designed alternative methods of engagement with eNav to increase the tool’s usability, accessibility, and impact for patients with diverse needs, including those with limited access to devices or the internet at home. Next, we will test the eNav intervention in a randomized controlled trial to evaluate the efficacy of the eNav website for improving CRC screening uptake among patients treated at FQHCs.

## Introduction

Colorectal cancer (CRC) is the second most deadly cancer, with more than 1.9 million new cases and 935,000 CRC-related global deaths annually [1]. Due to recent increases in early-age onset CRC, the United States Preventive Services Task Force lowered the recommended age for average-risk adults to begin CRC screening to 45 (previously 50) [2]. Several tests can screen for CRC, including at-home stool-based tests and visual tests (eg, colonoscopy) [3]. Recent data indicate that among individuals 45 years of age and older, more than 1 in 3 are not up to date with CRC screening [4]. Of concern, some patient populations are less likely to complete CRC screenings, including patients who receive care at Federally Qualified Health Centers (FQHCs). In fact, among patients treated at Health Resources and Services Administration–funded community health centers, including FQHCs, only 41.9% of adults aged 50-74 years have completed a CRC screening within the recommended time frame [5]. When including individuals aged 45-50 years, the CRC screening rates at FQHCs are likely even lower. In a national effort to reduce CRC morbidity and mortality, the National Colorectal Cancer Roundtable launched the “80% in Every Community” initiative with the goal of increasing the national CRC screening rate to 80% [6]. It is particularly important that we prioritize CRC screening among patients who are at greater risk of being unscreened, including the many patients treated at FQHCs.

Patient navigation is an evidence-based intervention designed to help individuals better navigate complex health care systems and overcome barriers to care [7]. More than a decade of clinical trials, including studies conducted in FQHC and community health care settings, have demonstrated that patient navigation can significantly improve CRC screening rates [8-16]. Although cost-effective [17,18], patient navigation typically requires economic resources (ie, hiring and training of staff), which can limit its ability to be widely integrated and sustained in standard clinical care, particularly in low-resourced or safety net health care settings such as FQHCs [16]. Digital patient navigation or patient navigation delivered via electronic media, is a novel form of patient navigation that holds promise for overcoming barriers to person-led navigation. Specifically, digital navigation may be a low-cost, accessible solution to improve CRC screening among patients treated at FQHCs. Importantly, patients can access digital patient navigation platforms from multiple locations and at convenient days or times. While digital patient navigation will likely not replace person-led patient navigation, it is possible that this intervention could reduce the workload and burden of patient navigators and providers through workflow changes and the optimization of technology [19].

Recent studies support the efficacy of digital patient navigation, especially SMS text message navigation, to improve CRC screening uptake [20-22], although results are mixed [23]. More broadly, other studies have examined the efficacy of digital health interventions to improve cancer screening uptake, including CRC screening [24]. More research is needed to better understand the impact of digital navigation interventions, especially those that incorporate novel features (eg, motivational support), can be accessed at flexible times and locations, and are offered in multiple languages.

In a collaborative effort to improve CRC screening uptake in the FQHC setting, our study team developed a novel digital navigation intervention. The eNav intervention includes a website and follow-up SMS text messages. The intervention is drawn from the Health Belief Model (HBM) [25] and aims to impact HBM-informed constructs (eg, perceived benefits and barriers, perceived susceptibility, and perceived severity) and, in doing so, improve CRC screening completion. Significantly, the study team conceptualized, developed, and iteratively user-tested eNav in close collaboration with community stakeholders. This ongoing partnership is integral in ensuring that an equity lens informs the eNav website and that it is appropriate for FQHC patients [26]. Our study team hypothesized that the eNav intervention will help improve CRC screening uptake among patients treated at FQHCs. The first step in this program of research was to conduct user-testing in order to examine the usability and acceptability of the website component of the eNav intervention with FQHC patients. This paper presents initial data from the iterative user-testing of the eNav website. This user-testing study is part of a larger research project aiming to test the efficacy of the eNav intervention in an FQHC environment within a randomized controlled trial (RCT).

## Methods

### Team Overview

The investigative team for this study represents a partnership between the Icahn School of Medicine at Mount Sinai, an academic medical center, and the Institute for Family Health, an FQHC network with clinics throughout New York City and the mid-Hudson Valley. Drawing from existing literature and the HBM, the investigative team developed the first iteration of the eNav website.

### Description of the First Iteration of the eNav Website

#### Intervention Overview

We designed the eNav website as a digital navigation tool for patients who are due or overdue for CRC screening and have an upcoming primary care appointment at an FQHC. The eNav website can be accessed on multiple digital devices (ie, computer, smartphone, and tablet) and at a time and place that is convenient for the patient. To maximize reach and impact, the website is offered in English and Spanish, can be accessed before and after the patients’ primary care appointment, delivers information over time (via text, graphics, and close-captioned videos), and allows patients to request a screening test. The eNav website contains the components explained next.

#### Information

On the primary landing pages, the eNav website offers information about CRC including (1) CRC and its risk factors, (2) polyps, (3) signs and symptoms of CRC, (4) the role of screening, and (5) the importance of screening for all people older than 45 years. The website also includes a “Frequently Asked Questions” page and an “Additional Information” page that provides more detailed information to help navigate patients to get screened including instructions on how to complete each CRC screening test and information about overcoming barriers to screening (eg, information about insurance coverage).

#### Decisional Support

Previous research has shown that CRC screening completion rates may improve when patients are given a choice of which test to complete [27]. As such, the eNav website includes a “screening options” page that provides information via text, images, and animated videos about 3 commonly used CRC screening tests: fecal immunochemical test (FIT), multitarget stool DNA test (FIT-DNA), and colonoscopy. The website also provides a table comparing the requirements for each test (eg, collecting a stool sample at home for FIT and FIT-DNA and going to a clinic or hospital for the colonoscopy procedure), acknowledging barriers patients may face for each test. Crucially, the website makes clear that for some patients, a colonoscopy may be the test recommended by their provider. Significantly, information about coverage of the costs related to each procedure is also included.

#### Motivational Support

An integral component of the eNav website is motivational support for completing CRC screening, offered through a “My Why” video that features patients from diverse backgrounds sharing their reasons for completing CRC screening. The eNav website encourages users to identify their own reasons for wanting to get screened for CRC.

#### Risk Assessment

Not all participants are eligible for stool-based testing. For example, some patients with symptoms (eg, rectal bleeding) or certain risk factors (eg, Lynch syndrome) may need a colonoscopy rather than a stool-based test. To facilitate conversations between patients and providers about CRC screening options, the eNav website includes a risk assessment that helps determine whether patients are eligible for stool-based testing. Although not a comprehensive risk assessment, the tool assesses whether patients have current physical symptoms that could necessitate a colonoscopy (eg, blood in the stool). They are also asked whether they have a personal or family history of conditions that can increase the risk of developing CRC (eg, Lynch syndrome). After completing the risk assessment, patients receive an output delineating their CRC screening options. If patients do not report any of the listed symptoms or potential risk factors, the eNav website offers patients a choice to request a stool-based screening (ie, FIT or FIT-DNA) or a visual screening (ie, screening colonoscopy). Of note, if patients are flagged as potentially ineligible for stool-based testing, eNav recommends that these patients talk to their primary care provider about the most appropriate screening option and displays a video of a provider discussing CRC risk factors. The results from the risk assessment are sent to the treating primary care provider and can be used to guide shared decision-making about CRC screening. The questions and responses for the risk assessment were adapted (with permission) from the Colorectal Cancer Alliance CRC screening quiz [28]. The content of the questions was also guided by the C5 Colon Cancer Prevention Risk Assessment and Screening Form [29]. Our investigative team and clinical partners, including family physicians and gastroenterologists, reviewed the final risk assessment.

#### Option to Request a CRC Screening Test

If patients are deemed potentially appropriate for stool-based testing through the risk assessment, they are prompted to select a CRC screening test—FIT, FIT-DNA, or colonoscopy. If a patient requests a CRC screening test, the treating primary care provider will be sent a message notifying them that their patient is interested in the selected CRC screening test. The treating primary care provider will then determine whether to place the order for the CRC screening test. Importantly, all patients, regardless of risk level, will have the chance to discuss CRC screening with their provider at their upcoming primary care visit.

#### Text Reminders + Instructions

The eNav intervention is composed of the eNav website and follow-up SMS text messages. In addition to viewing the website, patients will receive a series of SMS text message reminders to help navigate them to get screened for CRC. For example, patients who request a FIT test are instructed to pick up the FIT test at their appointment, are reminded to complete the test after their appointment, and are given information on how to receive another test, if needed. The SMS text messages also contain hyperlinks to different sections of the eNav website to help them effectively prepare for and complete the CRC screening tests (see Multimedia Appendix 1).

### Procedures

**Figure 1:**
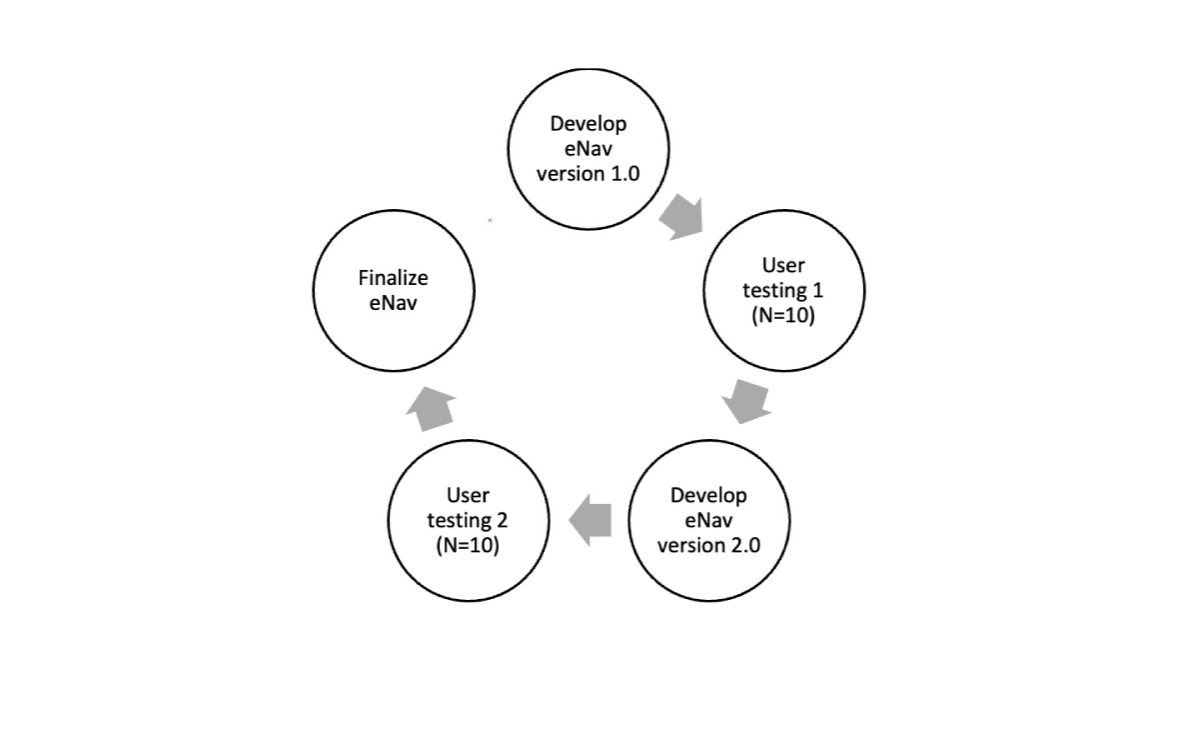
Iterative eNav website user-testing procedures.

As demonstrated in Figure 1, two user tests (n=10 participants per user test) were conducted to gather feedback on the eNav website from FQHC patients. The first user test was conducted in December of 2022 and the second was conducted in May of 2023. For each user test, study team members distributed flyers in an FQHC clinic waiting room and lobby to recruit patients to participate in the study. If patients expressed interest in the study, they met with a research coordinator in a private room to complete the informed consent process. After providing informed consent, patients were asked to view and interact with the eNav website on a study-provided computer or on their personal device (eg, smartphone). Participants engaged in a “think aloud” exercise, during which they were asked to provide their feedback, in real-time, on the eNav website. During the think-aloud exercise, study team members produced behavioral note summaries, including their observations of participants’ behaviors and any challenges interacting with eNav. After the think-aloud exercise, participants engaged in a brief qualitative interview to provide additional feedback about the eNav website (eg, suggestions for change). The think-aloud exercises and interviews were audio-recorded. Although the SMS text message reminders and instructions component of the intervention was not comprehensively user-tested, participants also provided insights on their preferred frequency of SMS text messaging. Finally, participants in each user test completed a questionnaire that assessed demographic information, technology and internet use, medical history (eg, history of having CRC screenings), and health literacy.

The study team used the results of the first user test to refine the content and improve the user experience of the website to create eNav version 2.0. Then, this second iteration of the eNav website was user-tested on a new set of participants, using the same procedures, to confirm that the new iteration of the app was both usable and acceptable. The results from the second user test were again used to refine and finalize the eNav website.

### Participants

Patients were eligible to participate in the user-testing if they were (1) treated at an FQHC, (2) aged 45-75 years, (3) English- or Spanish-speaking, and (4) able to provide consent. Patients were excluded if they were hearing or vision impaired. Participants who completed the first user test were not eligible to participate in the second. In total, the sample size for the first 2 user tests was 20 patients.

### Measures

#### Medical History and Demographics

A brief survey was used to assess participant demographics (eg, age, race, ethnicity, and sex), medical history (eg, previous CRC screening), and technology ownership and use. The survey questions were adapted from demographic surveys used in our team’s previous research and from questions from the PEW Research Center [30,31].

#### Usability

The System Usability Scale (SUS) [32] evaluated the usability of the eNav website. The SUS includes 10 items scored on a 5-point Likert scale (1=strongly disagree and 5=strongly agree). We adapted the language of the SUS from “this system” to “this website” so that it was applicable to eNav.

#### Acceptability

The Acceptability E-scale [33] assessed the acceptability of the eNav website. The E-scale is composed of 6 questions scored on a 5-point Likert scale. We adapted the language of the E-scale from “this computer program (ESRA-C)” to “this website.” Furthermore, we expanded the question, “How understandable were the questions?” to be, “How understandable were the questions about your health on the website (eg, risk assessment)?” and altered the item, “Was the amount of time it took to complete this computer program (ESRA-C) acceptable?” to be, “Was the amount of time it took to navigate this website acceptable?” We also shortened the question “How helpful to you was this computer program (ESRA-C) in describing your symptoms and QOL?” to “How helpful was this website?”

#### Health Literacy

To evaluate participants’ health literacy, we used 1 question from the BRIEF scale [34]. The BRIEF scale is composed of 4 questions, but previous research studies [35] have demonstrated that 1 screening question may be sufficient for detecting limited and marginal health literacy skills in clinic populations “How confident are you filling out medical forms by yourself?”

### Data Analysis

Thematic analysis [36] was used to analyze the qualitative data. Study team members transcribed the audio recordings of the think-aloud exercises and qualitative interviews. Then, the study team thoroughly reviewed the transcripts and behavioral notes. The data were first coded into 2 broad categories, based on the interview guide—“endorsed features” and “problems/suggestions for change.” After the first round of coding, the study team developed a coding scheme and codebook based on the “endorsed features” and “problems/suggestions for change” commonly identified by participants. Then, transcripts and behavioral notes were coded a second time, based on the codebook. All transcripts were coded by at least 2 independent coders. Once the coding was complete, themes were identified, reviewed, and defined. Given that the purpose of the study was to understand the participants’ perceptions of the intervention, our qualitative analyses focused on identifying semantic-level themes.

We applied descriptive statistics to analyze the medical and demographic information. To analyze the SUS score, we summed a contribution score for each item. Then, we multiplied the total score by 2.5 to produce an overall score ranging from 0 to 100. An SUS score greater than 68, the mean score, is considered acceptable [37]. To analyze the website’s acceptability, the scores on the Acceptability E-scale were summed to produce an overall score ranging from 6 to 30. According to the literature, a score of 80% or higher (total score of 24 or higher) is considered acceptable [33].

### Ethical Considerations

The study was performed in line with the principles of the Declaration of Helsinki. Study procedures were reviewed and approved by the Icahn School of Medicine at Mount Sinai’s institutional review board (STUDY-20-01888). Informed consent was obtained from all individual participants included in the study. All study data were stored in HIPAA (Health Insurance Portability and Accountability Act)-compliant secure servers and were deidentified prior to analysis. Participants were compensated with a US $50 gift card for their time and effort.

## Results

### Demographic Results for User Tests 1 and 2

We enrolled 10 participants in user test 1 and 10 participants in user test 2. See Table 1 for demographic information and medical history for both user tests.

**Table 1:** Demographic characteristics of user test 1 sample (n=10) and user test 2 sample (n=10) for eNav website user-testing study.

Demographic category		User test 1		User test 2
**Age (years)**				
	Range	52-69	54-74	
	Mean (SD)	61.3 (5.66)	62.8 (5.98)	
**Language, n (%)**				
	English	8 (80)	4 (40)	
	Spanish	2 (20)	6 (60)	
**Race, n (%)**				
	White	0 (0)	1 (10)	
	American Indian or Alaska Native	1 (10)	0 (0)	
	Black or African American	3 (30)	4 (40)	
	Other	6 (60)	5 (50)	
**Ethnicity, n (%)**				
	Non-Hispanic or Latino	6 (60)	4 (40)	
	Hispanic or Latino	4 (40)	6 (60)	
**Sex, n (%)**				
	Male	4 (40)	1 (10)	
	Female	6 (60)	9 (90)	
**Employment, n (%)**				
	Unemployed	8 (80)	6 (60)	
	Employed	2 (20)	4 (40)	
**Past completed colorectal cancer screening test, n (%)**				
	Colonoscopy	6 (60)	9 (90)	
	Fecal immunochemical test (FIT)	2 (20)	0 (0)	
	Multitarget stool DNA test (FIT-DNA)	2 (20)	0 (0)	
	Stool-based test, unsure which one	0 (0)	1 (10)	
**Family history of colorectal cancer, n (%)**				
	Yes	2 (20)	1 (10)	
	No	8 (80)	9 (90)	
**Marital status, n (%)**				
	Married or domestic partnership	3 (30)	4 (40)	
	Separated	2 (20)	0 (0)	
	Widowed	1 (10)	1 (10)	
	Single (never married or in a domestic partnership)	4 (40)	5 (50)	
**Highest level of education completed, n (%)**				
	<High school	2 (20)	3 (30)	
	High school (12th grade)	2 (20)	3 (30)	
	Some college	4 (40)	3 (30)	
	College	2 (20)	0 (0)	
	Missing	0 (0)	1 (10)	
**Health insurance, n (%)**				
	No insurance	0 (0)	4 (40)	
	Private health insurance	1 (20)	0 (0)	
	Medicare (including Health First)	1 (10)	0 (0)	
	Medicaid	4 (40)	3 (30)	
	Medicare and Medicaid	2 (20)	0 (0)	
	Other (Metroplus)	1 (10)	0 (0)	
	Other (United Healthcare)	1 (10)	1 (10)	
	Other (Undefined)	0 (0)	2 (20)	
**Estimated total household income (US $), n (%)**				
	Less than 10,000	1 (10)	0 (0)	
	10,000 to 19,999	0 (0)	1 (10)	
	20,000 to 29,999	1 (10)	2 (20)	
	30,000 to 39,999	0 (0)	1 (10)	
	40,000 to 49,999	0 (0)	1 (10)	
	50,000 or more	1 (10)	2 (20)	
	Prefer not to answer	7 (70)	3 (30)	

### User Test 1 Results

#### Technology Access and Use

Of the participants who completed the first user test, only 70% (n=7) had a smartphone. Of the respondents who reported not having or being unsure if they had a smartphone (n=3), 2 reported using no other devices at home and 1 used a tablet. Although 9 (90%) of the participants in the first user test accessed the internet at home, the quality of their internet was variable. Of the first user test sample, 1 (10%) reported not accessing the internet at home, 6 (60%) reported having slow internet speed, and 2 (20%) reported having interrupted internet. In fact, only 2 (20%) of the participants reported that they had no problems with their home internet.

#### Health Literacy

Regarding health literacy, all participants reported a certain degree of confidence in completing medical forms independently; 4 (40%) considered themselves “somewhat confident,” 1 (10%) said they were “quite a bit confident,” and 5 (50%) reported feeling “extremely confident” with the task.

#### Usability and Acceptability

The average SUS score for our first user test was 75.25, which is above the suggested benchmarks for usability [37,38]. The average Acceptability E-scale score for our first user test was 28.3, which is higher than the suggested benchmark for acceptability, and close to the maximum score of 30 [33].

#### Feedback From Think-Aloud Exercises, Behavioral Note Summaries, and Qualitative Interviews

In Table 2, we separated positive and negative feedback on the eNav website from user test 1 into the themes identified in the data.

**Table 2:** Positive and negative qualitative feedback on eNav website from user test 1 study sample (n=10).

Themes			Example quotes or behavioral observations
**User test 1: positive feedback**			
	**Content**		
		The content was interesting, informative, and important The content was easy to understand The website had diverse representation	“I like it. You can’t get no plainer than that. You explained everything. You know why you need to do it and how it needs to be done, you know, in all different kinds of ways...it’s very, very interesting, Like I said, there’s a lot of stuff I learned for myself just watching this, that I didn’t even know. I’m grateful that I took the time to watch it, and I guarantee that anybody that watches this, they’re going to learn a lot from it, and change their life.” “The fact that it is such a step by step showing the individual tests that are available and how applicable it is to each, how different it is to each individual...is great...” “This is wonderful. I like the different races in here to show...Beautiful, bravo, that video was outstanding. Outstanding in so many ways. Hitting the ethnic groups, you know, different cultures, everybody.”
	**Usability and acceptability**		
		The website was user-friendly and easy to navigate	“It is easy to navigate, and the videos are very attractive and educative.” “I believe that even the person that is not tech savvy will be able to...it’s pretty straightforward.” “It’s ideal for a person that’s busy on the go, so you are stopping for a minute, you’re going through a video, and you can fill out a form. One two three.”
	**Design**		
		The style and design of the website was appealing	“I like the website, and the color is perfect...the videos are very attractive and educative.” “It feels great...You know, the welcoming faces. They are not frowning or anything. They’re quite content, which is nice.” “I like the animated [videos]. I think it’s cute. It’s like it’s friendly.”
	**Motivation**		
		The website would motivate people to get screened for CRC^a^ Individuals wanted to share the website with others	“I’m interested more than ever before to get checked out.” “It’s really good, I like it, it will make you get less scared. And it helps you to be more, you know, let me go and do this, it is encouraging. Now I’m going home now to talk to my husband, because you know, he needed to do a colonoscopy...” “...this right here conveys the convenience and the ease of testing so that people won’t be scared of the process.”
**User test 1: negative feedback and suggestions for change**			
	**Content**		
		The content should include more information The content should use lay-language and avoid medical jargon The content should address ambivalence about completing CRC screening	“I think there should be a little more information about what is cancer...That’s what seems to be missing, you know what I’m saying? Because this right here also assumes the listener and the reader knows what cancer is” “What I want to know is like some people like to get the colonoscopy, right, you have to pay for it, you have to have the medical insurance, some people don’t have medical insurance, so how do they get it done if they don’t have medical insurance?” “What does [CRC] look like, how does it start? Where does it come from? How does it develop?...And there are food products that you eat, and you don’t know they’re harming you…I want to see how it is? how does it start?” Translated from Spanish: [“¿Cómo se ve, como empieza [el cáncer colorrectal? ¿De donde proviene? ¿Cómo se desarrolla? Y hay productos alimenticios que tú comes y no sabes que te están haciendo daño...Yo quiero ver ¿cómo es? ¿ Cómo empieza?”]
	**Usability and acceptability**		
		The website should be more accessible for those with limited technology skills or ownership Alternate versions of the eNav website should be offered	Not all participants requested a CRC screening test (behavioral observation) “Most people, a lot of people, like older people, they don’t have experience with the computer. To know more, they have to go on the computer, there should be other ways for them to learn about this...[Doing it on the phone] would be perfect.” “I am tech savvy to navigate through it, however, there are a lot of individuals that are not. And remember when you’re hitting the low-income areas, that’s a toughie right there. It would be great to put it in the hospital and have like some volunteers walk them through it.”
	**Design**		
		The style and design of the website should be bolder and more engaging	“You want to keep it comprehensive and concise, you know, appealing, you know, wonderful, friendly, you know? So this is just a little bland right now. It can be jazzed up a little bit.” “Maybe a little cute card at the end thanking them for that? You know, a very nice little card that opens, a virtual card, thanking them for caring, for caring about yourselves with something like that.”
	**Motivation**		
		The website should emphasize the urgency of screening for CRC	“Should be a little more dramatic, with more emphasis on the seriousness of symptoms...not enough drama here about the actual condition, the harmful condition like blood, vomiting and pain.” “I also think that you should put somebody that has cancer and somebody that don’t have the cancer, so you could compare how the how it looks...You know, some people need to get scared because a lot of people don’t do the test.”

^a^CRC: colorectal cancer.

#### Solutions to Identified Problems

Drawing directly from the qualitative and quantitative results of the first user test, we made the following changes to the eNav website.

##### Include Additional Information

To address patient queries, we added and expanded content (via graphics, texts, and videos) to provide more information about CRC and the different CRC screening tests.

##### Emphasize the “Request Test” Cue to Action

Some participants did not select a test, so we emphasized and highlighted the button to request a test.

##### Improve Accessibility of Technology

We removed functions that require an elevated level of tech literacy (eg, login page). Patients will also have the option to come into the clinic and receive assistance using a clinic-provided device to complete the risk assessment and request a test in advance of their primary care appointments.

##### Offer Engagement Boosters

Patients who do not complete the eNav risk assessment within 7 days of their primary care appointment will be sent a message with a link directly to the risk assessment and the “screening options” video (eNav lite). If patients still do not complete the risk assessment within 2 days of their appointment, they will be sent a message with the “screening options” video and a phone number that they can call to complete the risk assessment and request a test (eNav with assistance).

##### Enhance Style

We added bolder colors and included stock photos.

##### Improve Accessibility of Content

We made the font bigger and bolder.

##### Address Ambivalence

We added content to address patients’ ambivalence, particularly surrounding getting a colonoscopy. For example, we added references to resources about colonoscopy, a video about the colonoscopy procedure, and a video including a testimonial from a patient who had completed a colonoscopy.

##### Emphasize Urgency

We added a “five facts about colorectal cancer” section highlighting relevant incidence and mortality statistics. We also included a patient testimonial from a cancer survivor about their experience being diagnosed and treated for CRC.

### User Test 2 Results

#### Technology Access and Use

In the second user test, all 10 participants (100%) reported having a smartphone. Additionally, all 10 participants (100%) accessed the internet at home. Similarly, to the first user test, however, the quality of their internet at home was variable. In fact, only 4 (40%) reported having no problems with their home internet; 5 (50%) reported experiencing slow internet and 3 (30%) reported experiencing interrupted internet.

#### Health Literacy

In terms of health literacy, 1 (10%) participant considered themselves a little bit confident filling out medical forms by themselves, 1 (10%) said they were quite a bit confident, and 8 (80%) reported feeling extremely confident with the task.

#### Usability and Acceptability

After excluding 1 participant with a missing value, the average SUS score for our second user test was 75.28, which is above the mean SUS score or the suggested benchmark for usability [37,38]. The average Acceptability E-scale score for our second user test was 29.2, which is higher than the recommended benchmark for acceptability and near the maximum score of 30. Furthermore, both scores improved marginally between the first and second user tests; the SUS score increased from 75.25 to 75.28 and the Acceptability E-scale score increased from 28.3 to 29.2, confirming that the second iteration of the eNav website remained highly usable and acceptable.

#### Feedback From Think-Aloud Exercises, Behavioral Note Summaries, and Qualitative Interviews

In Table 3, we separated positive and negative feedback on the eNav website from user test 2 into the themes identified from the data.

**Table 3:** Positive and negative qualitative feedback on eNav website from user test 2 study sample (n=10).

Theme			Example quotes or behavioral observations
**User test 2: positive feedback**			
	**Content**		
		The content was interesting, informative and important The content was easy to understand The content had diverse representation and perspectives	“Yeah, I feel good. Health is important, and it’s important to know things like this.” “Well, I really liked it. You informed me. and also gave me more information about things, it has also given me more guidance on the things that I should look for...” Translated from Spanish: [“Bueno en verdad me ha gustado. Por qué me ha informado. y también más información de cosas, me ha dado más orientación sobre las cosas que yo debo de buscar...”] “Everything, I loved it! I liked the screening stories, and I also liked the information about food, diet, exercise and also the part about what we should do to prevent. Prevention. It is very good, because we as individuals can avoid that. Not smoking, not using alcohol and you can prevent it so you don’t get that cancer. It’s very interesting.” Translated from Spanish: [“Todo ¡Me encantó!, Me gustó la historia de la detección y me gustó también lo de la comida, la dieta, el ejercicio. Lo que uno debe hacer para prevenir. La prevención. Está muy bien. Porque uno puede evitarlo, no fumando, no usando alcohol y uno lo puede prevenir para que no le de cáncer. Está muy interesante.”]
	**Usability and acceptability**		
		The website was user-friendly and easy to navigate	“Because you see people talking about their story, sometimes we don’t like to read...” “I like the videos because if people don’t know feel comfortable with the computer, but with videos, they’re just watching them, and there are people who don’t know how to read or something and listen to it.” Translated from Spanish: [“Los videos me gustan, por qué si la gente no sabe mucho con la computadora. Pero con los videos, los está viendo, y hay gente que no sabe leer o algo y lo escucha.”] “That’s all easy to understand. I could see it here on my phone and I see it well.” Translated from Spanish: [“Todo eso está fácil de entender. Lo pude ver acá en mi teléfono y lo veo bien. ”]
	**Design**		
		The style and design of the website was appealing	“It’s very colorful. It’s not boring...bright colors are good.”
	**Motivation**		
		The website would motivate people to get screened for CRC^a^ Individuals wanted to share the website with others	“It’s good, gives you, you know, courage.” “This page I am going to share it with my daughters, my friends, so that they can also read it.” Translated from Spanish: [“Esta página se la voy a pasar a mis hijas a mis amistades, para que ellos también la lean.”]
**User test 2: negative feedback and suggestions for change**			
	**Content**		
		The website should provide more clarification about technical language, particularly in the risk assessment The content should include more information The content should use lay language and avoid medical jargon	What is Lynch Syndrome? Translated from Spanish: [“Qué es el Síndrome de lynch?”] “The only thing. FITb and FIT-DNAc, they need to explain, what is the difference between those two. Yeah, everybody knows what colonoscopy is. But they didn’t explain why the FIT and the FIT-DNA are different.” “I think [the doctor in the video] could have went into a little more detail...She said, if you have this if you had that...she could have said...well, maybe covered a little more on symptoms.”
	**Usability and acceptability**		
		Improve accessibility and usability for those with limited technology skills or technology ownership, particularly in the risk assessment Correct technical glitch	Not all participants requested a CRC screening test [behavioral observation] Some participants asked the research coordinators to show eNav to them because they were struggling to navigate the website and the risk assessment on the computer [behavioral observation] Generally, participants were more comfortable scrolling through the website and navigating the risk assessment on their phones [behavioral observation] “Why don’t you navigate this thing? Because I’ll just tell you what my answer is.”
	**Design**		
		The website should improve the style and increase the font size	“I feel like [the font]’s a little small. Translated from Spanish: [“Siento que [la letra] es un poquito pequeña.”]
	**Motivation**		
		The website should emphasize the urgency of screening for CRC	“I think [the website] could be more powerful.” “Sometimes it’s better to shock people into the reality of the condition...If you value your life, you have to [get screened].” “These people are a little too soft for the seriousness of what we are talking about.” “I mean you could...have a couple more stories. You know, other people to tell them what they went through with, that, you know...But maybe with the screening, more testimonials...and how they are doing now, like the survivor story, experience.”

^a^CRC: colorectal cancer.

^b^FIT: fecal immunochemical test.

^c^FIT-DNA: multitarget stool DNA test.

#### Solutions to Identified Problems

Since the goal of the second user test was to improve eNav in preparation for testing the website within an RCT, we implemented the following solutions to address the major problems highlighted by participants.

#### Include Additional Information

To address participants’ desire for more information on selected topics, we created a glossary with in-text pop-ups throughout the website to define medical terminology. We also bolded or highlighted information about symptoms and expanded the informational content included throughout the website (eg, about CRC disparities, CRC risk factors, and the differences between FIT and FIT-DNA).

#### Update Risk Assessment

We altered the risk assessment language (eg, the descriptions of CRC symptoms) to be more descriptive. We also added “don’t know” to the “none of the above” answer options. In addition, we changed the functionality of the risk assessment so that it is more user-friendly (eg, automatically directs patients to the top of the next question).

#### Emphasize Urgency

We emphasized the CRC survivor testimonial by placing it in a more prominent part of the website. We also added questions about patients’ own motivations for screening to the “Patient Stories” page.

#### Improve Content Accessibility

In order to improve accessibility, we moved videos to the top of the website pages and removed large sections of text.

#### Enhance Style, Design, or Usability

Since many participants seemed more comfortable on their phones, we improved the presentation of the eNav website on phones. We also increased the font size on the website.

#### Fix Technical Difficulties

Participants encountered minor technical difficulties (eg, an error page); we collaborated with the developers to fix these technical glitches on the back end of the website.

## Discussion

### Principal Findings

This study conducted iterative user-testing of a digital patient navigation tool, which aims to increase CRC screening uptake among patients receiving care at FQHCs. The eNav intervention, informed by the HBM, provides critical navigation support to patients due for CRC screening, including information, motivational support, decisional support, and reminders. The eNav website can serve as an information hub, which can be accessed multiple times at convenient times and locations. Significantly, patients’ risk assessment answers and, if applicable, test requests are communicated to the health care team so that patients’ engagement with eNav is integrated into their care. It was critically important to user test the eNav in an FQHC setting to ensure that the website was appropriate for the intended users. See Multimedia Appendix 2 for a screenshot of a page from the updated, post–user-testing version of the eNav website.

Overall, our study demonstrated that the eNav website is highly acceptable and user-friendly. In both user tests, the usability and acceptability scores (as measured by the SUS and the Acceptability E-scale) exceeded the suggested benchmarks. Furthermore, the scores remained above the suggested benchmarks in the second user test, after significant website changes to address earlier user commentary. Moreover, during both user tests, respondents described the website favorably. For example, many participants stated that eNav was motivating, interesting, informative, and user-friendly. In both user tests, participants suggested including more content on the website to ensure that it conveyed all necessary information about CRC screening and the seriousness of CRC. In addition, respondents gave suggestions on how to improve the website’s usability, accessibility, and overall appeal. We used participants’ qualitative feedback to inform modifications to the intervention, especially in terms of content, design, and style, to ensure that the website has maximum acceptability and usability.

A key takeaway from the user tests is that some participants lacked the digital access needed to interact with the eNav website at home. The literature shows that, although technology use has grown in the last decade, disparities in technology access and use remain [39]. For example, according to the PEW Research Center, as of 2021, 43% of families with household incomes less than US $30,000 in the United States do not have home broadband services and 41% do not have a desktop or laptop computer [39]. Indeed, 27% of adults in lower-income households report using the internet only on their smartphones [39]. In our sample, we found that there was demonstrated variability in access to and quality of technology. While almost all of our sample (95%) accessed the internet at home, the majority of participants in both user tests reported some problems with their internet, including slow internet speed and interrupted service. Beyond digital access, some participants explained that they were concerned that members of their communities, especially older individuals, would not have the skills needed to navigate the website on their own, particularly on computers.

To maximize accessibility, including for patients with lower digital access and skills, and to ensure we are not inadvertently widening disparities in CRC screening completion, we designed the eNav intervention to be device agnostic and require minimal technology skills. Based on the results of the user-testing, we made additional modifications to the eNav intervention in order to improve accessibility and usability for patients with limited digital access and readiness. First, we moved all videos to be easily accessible at the top of each website page. Furthermore, and most notably, we removed the login process from the website; patients are now able to access the website directly and only need to provide their information to complete the risk assessment. In addition, as previously explained, patients who do not engage in the eNav intervention will be offered engagement boosters called “eNav lite” (text-based) and “eNav with assistance” (navigator-assisted) that can help them request a CRC screening test. Finally, patients who do not have access to technological devices at home (eg, computer, smartphone, and tablet) or who would prefer not to interact with eNav at home will have the option to view the website in person, on a clinic-provided device. These modifications are important not only for patients who struggle with technology access and literacy but also for patients who may prefer simplified forms of communication. In sum, we made these changes, alongside the previously mentioned refinements of the content and design of the eNav intervention, to increase the usability, accessibility, and impact of the intervention for patients with diverse needs.

### Limitations

Since we recruited patients from an urban FQHC clinic, convenience sampling is a limitation of this study. Patients who chose to participate in our research study may not fully represent the FQHC patient population. Furthermore, all participants were older than the age of 50 years, so there was no representation from patients in the recently expanded age range for screening (ie, age 45-50 years). A third limitation is that all participants in both user tests had completed a CRC screening in the past, although 2 participants in user test 1 and 1 participant in user test 2 were due for screening at the time of recruitment. Including patients who had never completed a CRC screening would have strengthened the study. Included patients, however, were able to draw from their previous experience with CRC screening to provide valuable feedback. A final limitation of the study is that participants’ responses may have been impacted by social desirability. In particular, the participants viewed and interacted with the eNav intervention with a member of the research team present. The presence of the research staff, as well as the overall study context, may have influenced participants to respond in a favorable or positive way. In order to mitigate social desirability bias, we encouraged candor and explained that the team was specifically seeking negative feedback and suggestions for change.

### Conclusions

The iterative user-testing confirmed the overall usability and acceptability of the eNav website. Furthermore, qualitative and quantitative feedback from participants directly informed modifications to the intervention. The RCT will formally evaluate the efficacy of eNav for improving CRC screening uptake among patients treated at FQHCs.

## Appendix

Multimedia Appendix 1 Follow-up text messaging content and dosing for eNav intervention.

Multimedia Appendix 2 Screenshot of "Colorectal cancer" page on eNav website, including animated video.

## Abbreviations

CRC: colorectal cancer
FIT: fecal immunochemical test
FIT-DNA: multitarget stool DNA test
FQHC: Federally Qualified Health Center
HBM: Health Belief Model
HIPAA: Health Insurance Portability and Accountability Act
RCT: randomized control trial
SUS: System Usability Scale

## Data Availability

The data sets generated and analyzed during this study are not publicly available to protect patient privacy.
