# Supplementary figures and images for: The Development of a Digital Patient Navigation Tool to Increase Colorectal Cancer Screening Among Federally Qualified Health Center Patients: Acceptability and Usability Testing
Source: JMIR Form Res. 2024 Sep 25;8:e53224. doi: 10.2196/53224 (PMC11464930; doi:10.2196/53224)

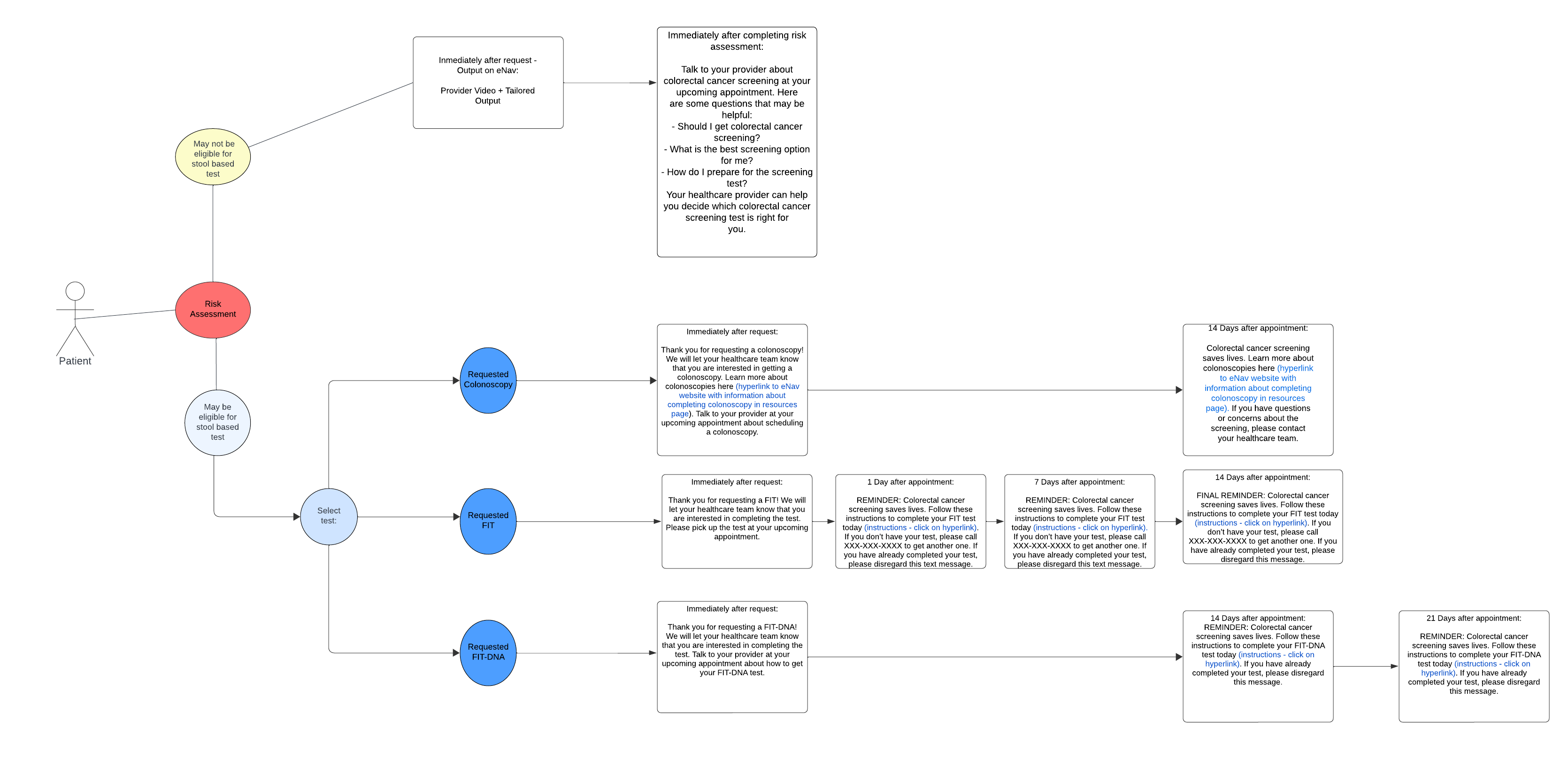

Supplement: Multimedia Appendix 1 [file formative_v8i1e53224_app1.png]

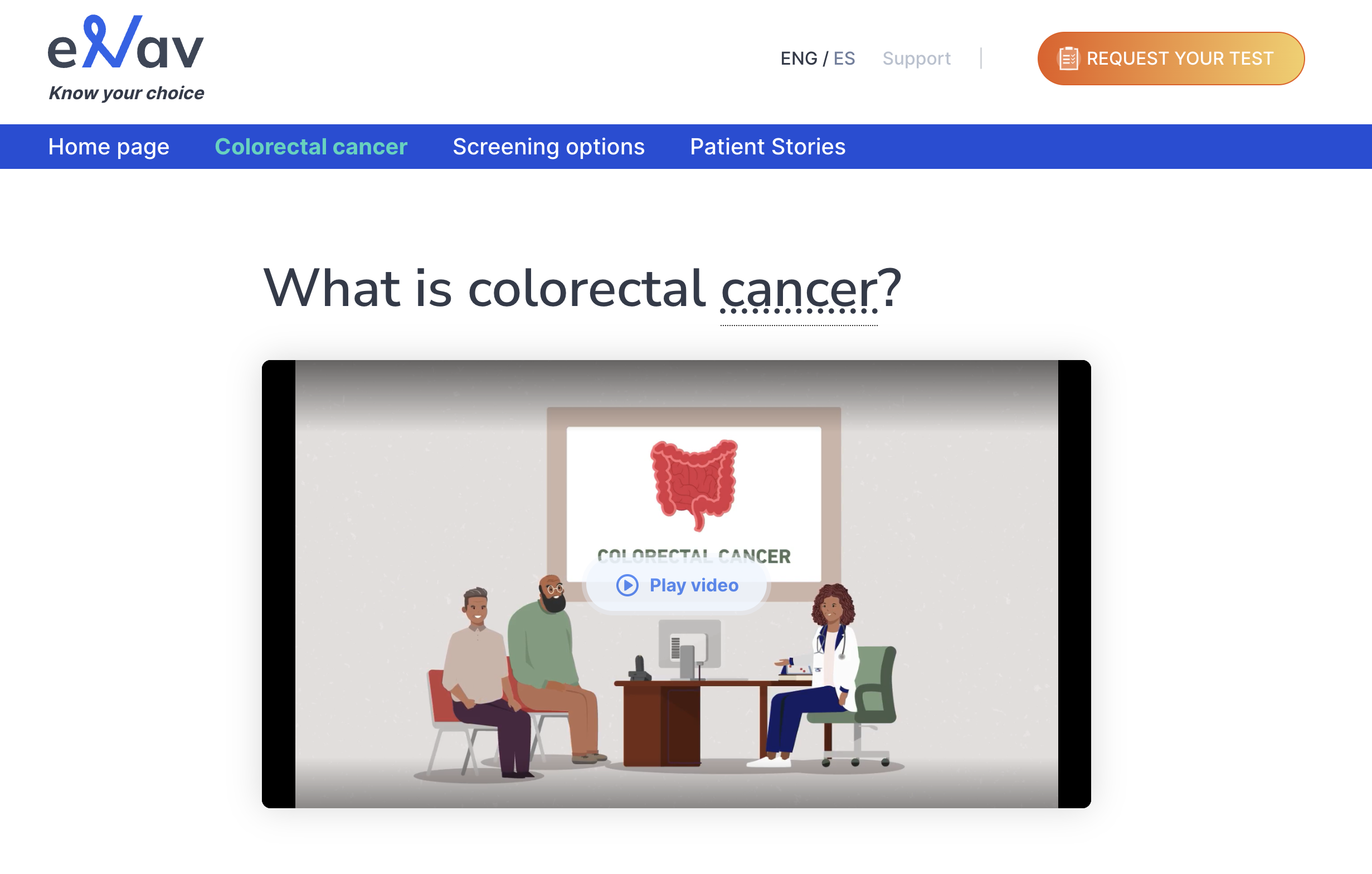

Supplement: Multimedia Appendix 2 [file formative_v8i1e53224_app2.png]
